# Older smokers could be the strongest supporters for U.S. government regulation of tobacco: a focus group study

**DOI:** 10.1186/1617-9625-11-17

**Published:** 2013-08-17

**Authors:** Valerie B Yerger, Janine K Cataldo, Ruth E Malone

**Affiliations:** 1 Department of Social and Behavioral Sciences, University of California, San Francisco, Box 0612, San Francisco, CA, USA; 2 Department of Physiological Nursing, School of Nursing, University of California, San Francisco, San Francisco, CA, USA

## Abstract

**Background:**

Targeting of marginalized groups with aggressive tobacco marketing has been identified as exacerbating health disparities. However, interpretation of such targeting by groups varies, from surprise and outrage to regarding such marketing as evidence of social legitimacy. We sought to learn how an often-overlooked marginalized group, older adults, would respond to industry documents offering evidence of tobacco company target marketing.

**Methods:**

We conducted 10 focus groups in California cities with older (≥50 years) smokers and former smokers. A set of previously-undisclosed tobacco industry documents related to target marketing was shown to the group in sequence. Audiotaped discussions were transcribed and data analyzed using qualitative approaches.

**Results:**

Responses to evidence of tobacco industry targeting varied, with some regarding it as exploitive and others as normal business practice. However, in most groups, discussions turned to government’s failure to protect the public—even though government action /inaction was not prompted nor addressed in the discussion documents.

**Conclusion:**

Given the Food and Drug Administration’s new authority to regulate tobacco products, these findings suggest that some of the tobacco industry’s “best customers” (older, established smokers and ex-smokers) may be strong supporters of government regulation of tobacco.

## Background

The absolute negative health burden from smoking is greatest for older adults (>50) [1]. Older adults are growing in number and are the least likely to quit of any age group, perhaps because they underestimate both the risks of smoking and the benefits of cessation [2]. While quitting smoking by age 50 halves the risk of lung cancer [2] and almost immediately decreases cardiovascular risk [3,4], older adults are often unaware of these benefits [5]. There are two primary reasons for these misperceptions; 1) the tobacco industry suppressed information on the impact of cigarette smoking on cardiovascular risk and the benefits of cessation for cardiac health [6]; and 2) the tobacco industry’s heavy targeting and marketing to older smokers [7] for both conventional and emerging tobacco products [8-10]. Tobacco companies use marketing to reduce perceptions of harm associated with tobacco use, increase perceptions that tobacco is socially acceptable, and ultimately encourage tobacco use [11-13]. Tobacco industry marketing exposure distorts perceptions about the availability, use, and risks of tobacco [14].

The targeting of marginalized groups (e.g., African Americans, Hispanic/Latinos, gays and lesbians, homeless, mentally ill) with aggressive tobacco marketing has been identified as exacerbating the health disparities that affect such populations [15-22]. Research suggests that perceptions of targeting by marginalized groups can vary from feeling exploited to surprise and outrage [23] or in the case of gays and lesbians, seeing target marketing as evidence of emerging social legitimacy [24].

As a group, smokers have become increasingly marginalized [25]. In particular, older adult smokers constitute a marginalized group that has often been overlooked in tobacco control efforts, but not overlooked by tobacco companies. Older adults often experience both social devaluation and poverty. Fixed incomes and financial market fluctuations contribute to income and social insecurity, regardless of employment history. Moreover, older adults attempting to preserve independence and quality of life frequently encounter a lack of necessary social services [26].

The tobacco industry has shown a keen interest in older smokers, exploring ways to attract older smokers and keep them from quitting [7]. Since 2005, adult smoking prevalence in California has declined across all age groups, but the prevalence of smoking among those aged 45 and older has declined at approximately half the rate of younger groups [27]. In earlier work, we demonstrated that tobacco companies aggressively targeted aging smokers [7]. This targeting included the development and promotion of ‘low tar’ cigarettes to discourage older smokers, a group who were beginning to develop health concerns, from trying to quit smoking. The purpose of this study was to explore how older smokers and former smokers would respond to evidence of tobacco industry efforts to target older smokers by sharing a set of previously secret but now publicly available tobacco industry documents related to targeting with focus group participants.

## Methods

### Sample

Between September 2008 and September 2009, we conducted 10 focus groups in California in the San Francisco and Los Angeles areas with older (≥50 years) smokers and former smokers. The sample (N = 76) was 40% Non-Hispanic Whites, 53% Blacks/African-Americans, and 7% Others. (See Table 1 for additional participant characteristics).

**Table 1:** Focus group participant characteristics

**(*N *= 76 )**	**FG 1**	**FG 2**	**FG 3**	**FG 4**	**FG 5**	**FG 6**	**FG 7**	**FG 8**	**FG 9**	**FG 10**
	**(*n *= 8)**	**(*n *=9 )**	**(*n *=9 )**	**(*n *=5 )**	**(*n *=4 )**	**(*n *=8 )**	**(*n *=11 )**	**(*n *=8 )**	**(*n *=5 )**	**(*n *=9 )**
Location	N. Cal	N. Cal	N. Cal	N. Cal	N. Cal	N. Cal	S. Cal	S. Cal	S. Cal	N. Cal
Age range, years	50–58	51–65	50–66	50–66	50–57	53–82	51–73	51–68	50–63	51–65
Gender Women Men	F-3	F-4	F-0	F-3	F-1	F-6	F-0	F-5	F-1	F-8
	M-5	M-5	M-9	M-2	M-3	M-2	M-11	M-3	M-4	M-1
Race/ Ethnicity*	5 Blk	9 Blk	7 Blk	1 Blk	4 Blk	4 Blk	6 Blk	2 Blk	2 Blk	8 Wht
	3 Wht		2 Oth	2 Wht		4 Wht	4 Wht	6 Wht	3 Wht	1 Oth
				2 Oth			1 Oth			
Status: Smoker Former smoker	6	7	9	4	4	2	6	4	2	3
	2	2	0	1	0	6	5	4	3	6

*Blk Black/African American, *Oth* Others (i.e. Asians, Hispanics, mixed race), *Wht* Non- Hispanic Whites.

Other Abbreviations: *F* female, *FG* focus group, *M* male, *N. Cal* Northern California, *S. Cal* Southern California.

Eligibility requirements were age 50 and above, a history of cigarette smoking, and an ability to speak and read English. To aid recruitment, we contacted local tobacco control coalitions in advance and drew on their assistance to identify recruitment options and venues for focus groups. Additionally, we contacted personnel at senior community, recreation, and health centers for assistance in posting recruitment flyers. Persons calling a toll-free number were screened by the project assistant who used a flow chart to check eligibility before scheduling them for a focus group. The study was approved by the University of California, San Francisco Committee on Human Research.

### Procedures

Focus groups provide rich descriptive data, and interactions in groups are a powerful means of exploring perceptions of those participating in the discussions [28]. All groups were conducted by an experienced moderator and co-moderator. The focus group protocol included: a) introductions, b) demographic survey, c) focus group discussion, and d) post-discussion questionnaire. The demographic survey included three smoking history questions; 1) Do you *now* (currently) use tobacco of any type (cigarettes, cigars, spit, chew, pipe, or other)? 2) What is/are (was/were) your preferred brand/s? 3) What is the usual number of cigarettes you smoke/smoked in a day?

We used an open-ended, low-moderator-direction approach to facilitate a discussion and to be consistent with the exploratory aims of the study [29]. To provide evidence of tobacco industry targeting of older people, a set of eleven internal tobacco company documents made publicly available through litigation against the tobacco industry were retrieved from the Legacy Tobacco Documents Library, located at http://legacy.library.ucsf.edu[30]. Selected documents represented examples of marketing plans, specific targeting strategies, and industry tactics to increase tobacco visibility and acceptance among older adult smokers (see Table 2). The investigators selected by consensus documents judged likely to a) provide evidence of industry targeting activities, b) contain information not previously known to participants, and c) stimulate discussion about the topic of industry targeting. We exercised intentional selection bias in this process and presented the documents in the same order to each group to ensure against unknown biases due to order of presentation that could shape the discussions differentially across groups. This procedure was used in previous work on which this project was modeled [23,24].

**Table 2:** Tobacco industry documents discussed

**Document title**	**Year**	**Document description**	**Highlighted quotation or comment**
October 1976 NFO Data – quitting/switching and smoker profiles [31]	1977	RJR report on quitting rates of low tar cigarette smokers	“NOW and CARLTON are not encouraging smokers to quit at an accelerated rate. There are no differences in quitting rates between ultra low ‘tar’ brands and other low ‘tar’ brands…We believe smoker retention rates will improve for NOW as it becomes more established and as we educate more smokers to the ‘lowest’ benefit of NOW, thus giving them a reason to stay with NOW.”
Attitudes towards smoking and health [32]	1978	Summary of smokers’ attitudes toward the tar, nicotine and health issue found in B&W collection	“The only smokers who could be considered to have finally and unequivocally rejected Low Tar cigarettes were all younger (under 25) men. At this age the idea of death or serious illness seemed unreal…But these smokers would age and would quite soon reach the stage in life when the health issue began to provoke anxiety.”
Philip Morris, USA marketing Research Department report on “quitters” [33]	1980	Internal PM memo presenting research findings on smokers’ responses toward quitting	“Low-tar smokers (and Ultra-lows) say they’ll quit more than smokers in general, but actually they quit less, especially Ultra-lows.”
Summary of Marlboro “low tar” in-depth interviews [34]	1980	Summary of market research presented by Leo Burnett Advertising to PM on consumer perceptions of a low tar Marlboro product	“In general, younger people are not as concerned about their health. Conversely[,] older individuals who had begun to take health issue more seriously appeared more favorable toward the ‘low tar’ concept.”
An exploratory study – mature smokers [35]	1987	Market research report presented to Lorillard Tobacco Company on how to effectively market to mature smokers, while focusing on product use, purchasing behavior and lifestyle changes	“Even though the term ‘low tar’ is being used here, it should be pointed out that almost none of the respondents know the specific tar count of the brands they smoke or of those they try. Instead, they say they ‘go by’ designations on the package (or in advertising), such as ‘low tar’ or ‘light.’
The NOW brand: recommended field marketing approaches for Americans aged 50+ [36]	1991	Market research report presented to RJ Reynolds (RJR) by Segmented Marketing Services, Inc (SMSi) on strategies and tactics to develop marketing activities for older Americans	“The rapidly growing size and increasing disposable income of older Americans makes this segment a prime target for many products and services.”
NOW cigarettes and the 50+ consumer [37]	1991	Market research report on consumers aged 50+ conducted by Wave, Inc for RJR	Shown as an example of market research on older smokers conducted by outside consultants
Philip Morris USA Seniors Project [38]	1992	A Philip Morris (PM) report on capturing senior smokers from competitive brands through direct mail	Shown as an example of market research on older smokers
CARLTON age profile [39]	1994	Internal American Tobacco Company memo discussing which age group would smoke low tar cigarettes	“America is Graying and the 50+ segment is growing rapidly…they’re entering the CARLTON Zone…As smokers age, they tend to migrate to Ultra Low Tar.”
Baby boomer analysis [40]	1995	Internal Brown and Williamson (B&W) memo discussing the data on the demographics of aging “baby boomers”	Shown as an example of industry interest in the aging baby boomer market
CARLTON bond direct mail [41]	Date Undisclosed	Description of B&W direct mail program	“Smokers collect UPC’s to earn the $50 Bond. In essence, we have locked up our smokers for a full 8-10 weeks. Most vulnerable outswitchers get the Bond for 8 carton UPC’s. All other smokers …10 carton UPC’s. Start of New Year is a vulnerable time for smokers…when they might reconsider their brand choice.”

*Source:* Legacy Tobacco Documents Library, http://legacy.library.ucsf.edu.

Documents included reports of field marketing research conducted by advertising firms for tobacco companies, which revealed that older smokers were concerned about the effects of smoking on their health, whereas younger smokers were not. Other documents elucidated why older smokers were targeted with advertisements promoting low tar cigarettes as a “healthier” alternative to regular cigarettes (despite industry knowledge that low tar cigarettes did not help smokers to quit). Key passages were highlighted to launch discussions.

Participants were asked to respond to questions from the following domains: tobacco industry targeting of mature smokers and aging Baby Boomers; developing and promoting “low-tar’ cigarettes for the mature market; promoting the choice of “low-tar” while knowing that older smokers did not understand low-tar; using multiple strategies to discourage quitting among older smokers; and aggressively marketing to mature smokers. Using the protocol as a guide, the moderator allowed the group dynamics to shape the direction of the ensuing discussion and encouraged participants to engage with one another in a broad discussion on the topic of tobacco industry targeting. We provided light refreshments to study participants and reimbursed them $40 for their time. Groups were audiotaped and transcribed verbatim by a professional transcription service.

### Data analysis

Transcripts were reviewed and analyzed by the research team using an iterative approach in which each group transcript was read independently several times. Major themes were identified and discussed. Coding criteria were validated and refined through line-by-line review and discussed among the co-authors [28]. Once major coding categories were established, coding of all transcripts was completed by a trained graduate student research assistant. Transcripts were categorized by assignment of inductively-developed thematic codes to segments of text. Next, all segments assigned to codes were reviewed by members of the research team to identify recurrent themes and patterns common to the groups and to identify text segments that illustrated representative or contrasting examples. These segments, in turn, were further analyzed to clarify the key findings discussed below, using an interpretive approach focused on elucidating the meanings of participants’ responses [42-45]. When identified, discrepancies were discussed until consensus emerged. The NVivo8 software program [46] was used to facilitate data management and analysis.

## Results

Documents discussions elicited various responses from participants, including expressions of anger, intimidation, and surprise about the tobacco industry’s targeted marketing of older individuals and attempts to keep them smoking regardless of their age. Each document sparked lively discussions, which often expanded to include participants’ personal experiences. Though several themes emerged from discussions, this paper will focus on five themes that provide insight into areas related to older people that have not previously been reported in the literature. These themes are responses to target marketing, choice, and the broad theme of responsibility, which we further sub-coded into personal, tobacco industry, and government responsibility. The themes of target marketing, choice, and tobacco industry responsibility were discussed in all ten focus groups, as would be expected given the selection of documents reviewed; personal and government responsibility came up in nine and eight groups, respectively, a key finding since these topics were not addressed in the documents nor prompted by the moderator.

### Segmented marketing to older smokers

A copy of a field research report that Segmented Marketing Services, Inc. (SMSi) conducted for RJ Reynolds demonstrated how the tobacco industry valued older smokers as reliable and loyal customers [36]. Because this was the first document presented to each group, the highlighted quotations in the SMSi document elicited an initial response of surprise. Participants were unaware that, as a group, older adults had received special attention from the tobacco industry. This document showed that the tobacco industry was interested in older people because the number of older Americans was “rapidly growing” and the “increasing disposable income of older Americans makes this segment a prime target of many products and services” [36].

The report’s mention of “increasing disposable income” led to emotionally charged responses from participants, who took issue with this characterization of older smokers for marketing purposes. “Where do we have an increasing disposable income? We have less income…our income is so limited now…it’s very insulting to me.” (M, FG-2) The “insult” in this instance appeared to be the marketers’ misapprehension of the financial status of many older smokers. Given that smokers generally tend to be poorer than nonsmokers [47], this perception reflects the likely experience of many older smokers who may be living on fixed incomes or may be disabled and unable to work. Though there might have been some conflation of the idea of “disposable income” (money left over after paying essential expenses) with their own social “disposability,” the insult here appears to lie in a stereotyped version of older people that did not reflect participants’ experience.

A participant in another group also characterized the same document as an “insult,” but for a different reason:

“I find it to be insulting that…as we grow older… they want to keep us smoking until the day we die…It makes me feel like the cigarette industry is looking at me as being disposable and they’re going to keep me hooked as long as they can… until the day I drop dead of emphysema or lung cancer. .. I find that insulting. (M, FG-1)”

It is evident here that the “insult” is experienced as exploitation, in which older smokers are used up and then discarded and that there is something disturbing about continuing to market deadly products to those who are advanced in age.

However, not all participants found the tobacco industry’s targeting of older people offensive. “Well, first thing, I’m a smoker… I wish I didn’t… But considering that I do smoke, that doesn’t bother me that they’re watching or even advertising.” (M, FG-9) This sentiment was shared by several participants. Though most participants had previously been unaware of the tobacco industry’s intentional targeting of older people, there was a general acceptance across all groups that these activities could be considered “business as usual,” because the tobacco companies’ main objective was money-making. “The tobacco companies, they had medical evidence that smoking is bad for you…[they’re] not in the business to worry about your health [but] to move the product.” (M, FG-3) Participants frequently spoke of not being surprised by the industry’s focus on older smokers, because “part of their job is trying to make all the money they can.” (M, FG-7)

### Choice

These discussions were often followed by discussions about the role of personal choice. “Well, I believe that businesses have the right to earn money… it’s my choice to smoke. Any way that they can target [consumers] to get that money, God bless them.” (M, FG-4) Similar conversations arose in eight other focus groups. For example, after describing her decision to smoke as based solely on her personal choice to do so, a female participant met resistance from a male participant, who questioned whether she was able to make a choice if she was uninformed about the addictiveness of the product. “You weren’t given the choice to make a responsible decision,” he argued, “Because you didn’t know what you were smoking.” (M, FG-6)

However, consistent with studies that have found older smokers/lung cancer patients to engage in self-blame [48-50], others insisted that smoking was solely their own choice: “I can’t blame the tobacco companies; it is still me. Because I still have the ultimate choice.” (M, FG-2) “I think, ultimately, the responsibility is ours… Ultimately, it wasn’t their [tobacco industry] responsibility. It was ours.” (F, FG-6)

A dialogue between two male participants in FG-2 illustrates the tension in trying to decide whom to hold accountable: the tobacco industry or the individual: “I know better, and I smoke because I like it… But I don’t like being manipulated.” (M1, FG-3) “How can you really say that you have been manipulated when you have a choice?” (M2, FG-3)

It was generally accepted that because tobacco companies have a product to sell, they will do whatever it takes to sell more of it. “I’m not really surprised because it’s a billion-dollar industry, and they have to make their money, whatever they have to do by any means necessary. It’s not a good thing, but that’s just the way it is.” (M, FG-1) This understanding of the reasons for aggressive industry practices, however, did not mean that they were condoned. Rather, participants pointed out the need for stronger government regulatory action.

### Roles of government

Though government’s role was not addressed in the discussion documents presented to any of the groups, participants frequently raised on their own the issue of the government’s responsibility for protecting consumers and holding the tobacco industry accountable for the marketing of deadly products. Focus group participants honed in on government accountability as discussions developed around tobacco companies’ business activities and why there were no regulations or regulatory actions against such activities. Several participants displayed skepticism, however, about whether the government could or would intervene to protect the public from the tobacco industry. Whenever this topic came up, participants were quick to respond with reasons for the government’s permissive allowance of industry activity. One participant blamed “money” for the government’s relaxed stance on regulating the tobacco industry. “Why doesn’t the government just say, ‘Okay. Let’s just stop this. Ban it. Cut it out totally’? It goes back to one thing…money.” (M, FG-5) The nature of these perceived monetary disincentives for government regulation, however, appeared vague.

There were concerns that the government would not enforce existing regulations because it was somehow colluding with or profiting from the tobacco industry. “We realize that the tobacco company has a plan, like [making] a dollar. That the Surgeon General, our government won’t do anything because they got their hands in it. That’s why they’re not going to say anything.” (M, FG-2). The government’s role in protecting the industry’s profit continued as a focus of discussion in the same group:

“I guess they just all got together to see, you know, what was going to be profitable, and what were they going to have to do to continue their [products] to keep [them] flowing, you know; so they could keep getting the income that they were receiving from it, not really caring about whose life they were destroying, if they cared at all… People that’s up high that’s making all the money, they don’t care about nobody down here, as long as their money keeps coming through, you know?… It’s a sad situation… You know what I mean… it’s just sad. (F, FG-2)”

The discussion continued as others chimed in about mistrust of the government’s commitment to protect public health over and above the protection of industry profits. “I wouldn’t put that past our government. As long as [it] can get a profit, [it] will continue to let this go on.” (M, FG-2)

“And they’re sitting around watching millions and thousands of people die every year from lung cancer, some kind related to tobacco -- some kind of diseases.... It’s funny how our [government] don’t stop this horrible crime that’s being committed by Philip Morris products, it’s a crime…As long as there’s money, [the government doesn’t] give a damn. (M, FG-2)”

A similar discussion took place in another focus group: “[Tobacco companies faced] some big lawsuits, and they lost, whatever. But, they still continue to sell cigarettes. If people in high places were so concerned, cigarettes would be banned… it’s too much money with it.” (M, FG-5)

### Legality does not absolve government of responsibility

Government’s logic in sorting out which types of “bad behavior” should be sanctioned versus tolerated was a source of bafflement. For these participants, the “legality” of tobacco did not absolve government from its obligations to protect the public by prosecuting not only those who committed acts of immediate physical violence, but those committing acts of slower and more hidden structural violence, addicting consumers to deadly products.

“But what I’m saying is how can they get upset because people are shooting people, if these people are basically killing me, just slower? They’re still taking my life and the government is aware that it’s taking my life, but it’s okay? (F, FG-1)”

For many participants, the apparent inconsistency of prosecuting people for illegal drugs or gun violence while allowing tobacco companies to continue making a profit from products that cause much greater levels of harm was inexplicable. Most participants were unaware of the United States Department of Justice lawsuit against the tobacco industry, in which the major tobacco companies were adjudged to have committed fraud and racketeering [51].

“But, the government knows that these tobacco growers are growing the tobacco, which is killing people. So, why can’t [the government] in turn prosecute the tobacco [company executives] like they’re doing [with] the drug dealers?… What’s the difference? Because [the tobacco companies are] white collar and [the drug dealers are] “street”? (F, FG-2)”

“It’s intentional murder. I don’t care how you put it…they done gave us something that’s the most addictive thing there is, and they knew it. (M1, FG-5)”

Though they displayed only a vague awareness of legal challenges tobacco companies faced and industry losses in court, participants questioned why there were no visible consequences or penalties for ongoing promotion and sales of cigarettes. Regarding the government’s obligation to protect its citizens, one participant asked, “-- why tobacco is on the market when it’s obvious that it’s going to kill you?” (M, FG-8)

“Because the United States government sits there and allows them to do it…I think all of what we do is based on being informed or not being informed. And we all, as a population -- it doesn’t matter what class you’re in – generally if you are not informed, misinformed, or targeted as this…is something to the company’s benefit. (M2, FG-5)”

### Public protection

The failure on the part of the government to intervene more aggressively came up in another group: “The Surgeon General says that these could be a hazard to your health, but they didn’t say it caused cancer…they did not tell you that directly…because the United States government [only allowed] them to say so much.” (M, FG-5) The government, in the views of these participants, should not continue to tolerate “business as usual”. “I just don’t think that [selling tobacco is] honest business. I think it’s very dishonest and very deceitful the way [the tobacco companies] do it, the way they market and study the different age groups. And then the government goes along with this?” (F, FG-2)

In one instance, a participant spoke approvingly of how the government had successfully intervened to reduce the public’s exposure to secondhand smoke in restaurants and other public venues. “I’m so happy that the government has stopped the secondhand smoke in restaurants and out in the public.” (M, FG-7) Participants in another group pointed out how the government had sometimes acted to protect the public in the past:

“I think Vantage even went as far as to send out sample packs of Vantage cigarettes in the mail. Because you would get three cigarettes in a little, bitty [packs]…But I think the government stopped that. The companies weren’t allowed to mail unsolicited [cigarettes]. (M, FG-6)”

This discussion about the government interrupting the mailing of free samples of cigarettes also took place in other focus groups. One male participant reminisced and pointed out that the government took some action, but implied more action needs to be taken.

“You know like you said, back in the 50s and 60s you [would] see all kinds of commercials for cigarettes. The United States government said it was bad for your health. That’s why they cut the [television] commercials out, right? [But,] they put them in magazines and stuff like that, and billboards. (M, FG-5)”

## Discussion

In this study, as shown in previous work [23,24], there were mixed views on how the phenomenon of tobacco industry targeting should be understood. While some participants largely regarded targeted marketing of tobacco as unwelcome exploitation, others saw targeting as merely representing business as usual, forwarding a view of smoking as an individual choice. However, and unique in the literature to date, many participants in our groups appear to have resolved this ambiguity by focusing instead on the role of government in protecting public health.

An important and timely finding from this study is that this sample of older smokers and former smokers in California believed that the government should take more direct action to protect the public from tobacco industry marketing of tobacco products. Although older smokers are often omitted from tobacco control research, these findings suggest that older adults could be strong supporters of the government’s efforts to regulate tobacco products. The fact that groups independently raised this issue without prompting suggests it has salience for them independent of the targeting documents they were asked to review. These findings should be investigated in larger representative samples.

The Family Smoking Prevention and Tobacco Control Act of 2009 [52] gave the FDA authority to regulate the manufacture, distribution, and marketing of tobacco products to protect public health. However, its focus is primarily on youth and the “ushering in [of] a new era of tobacco control by recognizing that almost all new users of tobacco products are under age 18.” The aim of the Act is to “curb the trend of new users becoming addicted before they are old enough to understand the risks and ultimately dying too young of tobacco-related diseases [52].”

The prevention of initiation of tobacco use by youth is an indisputable public health priority. However, the new FDA mandate includes the protection of all groups, including older adults. FDA’s authority to prohibit “reduced harm” claims including “light,” “low,” or “mild” descriptors is an important first step in protecting older adults, since “low tar” cigarettes were developed in response to the health concerns of older smokers, despite industry knowledge that such products had no health advantage and did not help smokers quit [7].

In terms of protection for all US citizens, including the disenfranchised and vulnerable, the federal government has made steps both forward and back. Three important steps forward include offering comprehensive quit-smoking benefits to millions of federal employees and their families; partial reimbursement by the federal government for quit-smoking counseling services for Medicaid enrollees (i.e., quit lines with state toll-free numbers); and the Affordable Care Act requirement that private insurers cover tobacco cessation services without cost sharing under the “Evidence-based screenings and counseling” category.

In a now-thwarted attempt at a step forward, the FDA unveiled graphic warning labels for cigarette packs, which were supposed to include the national quitline telephone number, 1-800-QUIT-NOW. However, in February 2012, the District Court in Washington ruled that the new graphic warning labels violated the First Amendment because the mandated graphic warnings went beyond factual disclosures and would force tobacco manufacturers to adopt the government’s anti-smoking message, exceeding the government’s legal authority [53]. In March 2013, the FDA announced that instead of appealing this ruling they will “undertake research to support a new rule-making consistent with the Tobacco Control Act [54].”

Consistent with all focus group studies, we note several limitations. First, the convenience sample of focus group participants, although relatively diverse, cannot be considered statistically representative of all older smokers and former smokers. The data were all collected in California, a state with a strong tobacco control program focused on norm change and tobacco industry denormalization [55-57]. California also has a strong tobacco control regulatory climate, demonstrating an active government role, with generally strong support for tobacco initiatives. External trends in social perceptions about government and corporate behavior might have influenced discussions.

## Conclusion

This study’s key finding was that older smokers appeared impatient with the pace of tobacco control reform, asking why the government did not do more, such as ban cigarette sales. Given the context of FDA regulation, which represents the most promising development at the federal level in many years, this finding lends support to the idea that the public may be ahead of policymakers in seeking to end the tobacco epidemic. Our findings suggest that some of the tobacco industry’s “best customers” would welcome government action and would regard it as consistent with government policies to address other harmful products and activities.

## Competing interests

VBY served as an expert witness in a deposition against the tobacco industry. JKC declares that she has no competing interests. REM owns one share each of Philip Morris International, Philip Morris USA, and Reynolds American tobacco company stocks for research and advocacy purposes.

## Authors’ contributions

VY collected data, provided input on the analysis and interpretation of data, and drafted the manuscript. JC made substantial contributions to the study’s conception and design, collected data, and assisted in writing the manuscript. RM contributed to the study’s conception and design, provided input on the analysis and interpretation of data, and assisted in writing the manuscript. All authors read and approved the final manuscript.

## Acknowledgment

The authors would like to acknowledge Andrea Corage Baden for her contributions to data coding.
